# Enhancing healthcare consensus mechanism - A reputation integrated variant of PBFT (BR-PBFT)

**DOI:** 10.1371/journal.pone.0336039

**Published:** 2025-11-11

**Authors:** Shamsudeen Sajna, Manu J. Pillai, Ginu Rajan

**Affiliations:** 1 Thangal Kunju Musaliar College of Engineering, Affiliated to APJ Abdul Kalam Technological University, Kollam, Kerala, India; 2 Centre for Engineering Research in Intelligent Sensors and Systems, Cardiff School of Technologies, Cardiff Metropolitan University, Cardiff, United Kingdom; Zhengzhou University of Light Industry, CHINA

## Abstract

In the rapidly evolving healthcare landscape, adopting blockchain technology requires an optimal consensus mechanism to ensure security, efficiency, reliability, and scalability. This paper examines various consensus methods, including PoW (Proof of Work), PBFT(Practical Byzantine Fault Tolerance), its variants, and node reputation management techniques such as the Beta Reputation Model and EigenTrust score, to determine the most suitable approach for healthcare applications. Among the analyzed algorithms, SBFT (Scalable Byzantine Fault Tolerance) emerges as a strong contender due to its fault tolerance and effectiveness in large-scale environments. Building on these insights, we introduce a light weight consensus BR-PBFT(Beta Reputation integreted PBFT), a novel PBFT variant that integrates a beta reputation scoring system and Verifiable random functions (VRF) for more reliable node selection. This enhancement strengthens trust in the network and mitigates malicious behavior by dynamically adjusting node selection and consensus processes based on reputation metrics. Preliminary evaluations indicate that our PBFT variant built on reputation significantly improves CPU consumption and memory efficiency, offering improved reliability, scalability, and performance. These advancements position BR-PBFT as a promising state-of-the-art solution for secure and efficient blockchain implementations in healthcare.

## 1 Introduction

The advent of blockchain technology has revolutionized various industries, particularly through its application in cryptocurrencies and other major industries. Its decentralized ledgers provide robust mechanisms for ensuring transaction trust and security, underpinning the value and functionality of digital currencies like Bitcoin and Ethereum [1]. The immutable and transparent nature of blockchain records has made it an attractive solution for many sectors. Blockchain technology has transformed various industries by offering decentralized, secure, and tamper-resistant data management solutions. However, the requirements that make blockchain technology suitable for cryptocurrencies do not seamlessly translate to other industries, such as healthcare. Managing health records with blockchain requires a more nuanced approach considering factors such as response time, reliability, and scalability [2].

The adoption of blockchain in healthcare is driven by the need for secure and efficient data sharing between multiple stakeholders, including hospitals, patients, insurance providers, and regulatory bodies. A key challenge in healthcare data management is ensuring trust and data integrity without relying on centralized authorities. Conventional data-sharing models often face issues such as single-point failure, data silos, and lack of real-time access, leading to inefficiencies and security risks. Blockchain addresses these concerns by offering decentralized trust, tamper-proof records, and transparent access control.

The effectiveness of blockchain in healthcare heavily depends on the underlying consensus mechanism, which determines how transactions are validated and added to the ledger. There are several consensus mechanisms on blockchains, such as those applicable in different use cases based on their utility. However, selecting an optimal consensus mechanism remains a significant challenge, as traditional approaches such as Proof of Work (PoW) and PBFT have limitations in terms of energy efficiency, scalability, and trust management.

An effective consensus mechanism must balance security, efficiency, and fairness, ensuring that transactions are validated by trusted nodes while maintaining high throughput and low latency. PoW, though secure, is computationally intensive and unsuitable for real-time healthcare applications. PBFT, a leader-based approach, offers better efficiency, but lacks a built-in trust model, making it vulnerable to malicious nodes and potential centralization. To overcome these limitations, an optimized consensus mechanism is needed: one that integrates trust-based node selection, computational efficiency, and fairness in leader election.

In addition to consensus algorithms, trust management is crucial in healthcare blockchain networks [3]. This research explores the Beta Reputation Score and Eigen Trust score as methods for assigning weights to nodes based on their historical behavior and trustworthiness [4]. By prioritizing interactions with reliable peers in the network [5] Beta Reputation System can mitigate the risk of data tampering or malicious attacks [6]. Furthermore, leveraging VRF adds a layer of security and randomness to the node selection process, ensuring a fair and unbiased consensus mechanism [7]. This integration of reputation scores and VRF not only enhances the resilience of healthcare blockchain networks but also fosters greater confidence among stakeholders, including patients, healthcare providers, and regulatory bodies.The rising adoption of blockchain in healthcare highlights its potential to address inefficiencies and enhance patient outcomes. As these initiatives scale, they can drive innovation and improve care quality. This study focuses on bias, trust, and efficiency, emphasizing the need for advanced, lightweight consensus mechanisms tailored to healthcare.

### 1.1 Background and key concepts

The healthcare industry faces persistent challenges in data security, interoperability, and trust management among stakeholders as discussed earlier. Existing electronic health record (EHR), IoMT (Internet of Medical Things) and healthcare managemnet systems often suffer from centralization risks, data breaches, and inefficiencies in information exchange. Blockchain offers a promising solution to these issues by enabling a decentralized, immutable, and transparent data-sharing environment. In IoMT, blockchain enhances device authentication and secure health data transmission, ensuring patient privacy [8]. Recent advancements in blockchain-based healthcare solutions have led to pilot projects to secure patient data exchange and improve care coordination [9]. Governments and regulatory agencies are testing blockchain for compliance tracking, such as verifying the provenance of pharmaceuticals and medical devices to enhance safety and authenticity.

Despite its benefits, healthcare systems still face persistent challenges, including data security risks, interoperability barriers, and delays in accessing EHRs [10]. Centralized EHR systems remain susceptible to breaches and inefficiencies in data sharing. Therefore, a tailored consensus algorithm is required to ensure data integrity while optimizing performance for healthcare applications.However, choosing the best consensus mechanism remains a big challenge, as older systems have limits in terms of trust management.

Blockchain’s immutability, decentralization, and controlled access provide a potential solution; however, some existing consensus mechanisms introduce latency and scalability issues [10]. As discussed, PoW provides high security and it is computationally expensive and energy-intensive, making it unsuitable for real-time healthcare applications and PBFT, an alternative designed for permissioned networks, improves efficiency but lacks a robust trust model, making it vulnerable to malicious node behavior and centralization risks. While cryptocurrencies rely on consensus mechanisms like PoW and Proof of Stake (PoS) for decentralization, healthcare blockchains prioritize trust, scalability, and regulatory compliance, which necessitate the development of an improved consensus mechanism that balances security, efficiency, and trust. On the other hand, among the traditional consensus mechanisms, PBFT provides low energy consumption and quick transaction finality, making it preferable for healthcare applications. To address the concerns discussed above, reputation-based trust models and cryptographic randomness mechanisms can be proposed to improve fairness, security, and efficiency.

Several variants of PBFT have been introduced to enhance scalability, efficiency, and security, such as SBFT, which reduces communication overhead using leader-based message aggregation; Honey Badger BFT (HBFT), which improves asynchronous consensus under high network latencies; and PBFT++, which optimizes view change mechanisms and batch processing to improve transaction throughput [11–13]. These variants demonstrate performance improvements but still lack a comprehensive trust-based approach that BR-PBFT aims to integrate. Therefore, to address this, trust management mechanisms such as the Beta Reputation Score (BRS) and EigenTrust, which can assign trust scores to nodes based on historical performance and ensure only reliable nodes participate in consensus, can be considered. Reputation scores plays a crucial role in blockchain by addressing trust challenges in decentralising systems [14].

The Beta Reputation System operates on the principles of Bayesian inference. This statistical technique updates beliefs about the likelihood of an event occurring based on prior knowledge and new evidence. In the context of blockchain, this calculates reputation scores for network participants by aggregating feedback from various sources, such as user reviews, transaction history, and interactions within the network [15]. The calculation involves probabilistic models that consider the reliability of the information sources and the consistency of feedback over time. By analyzing the credibility and consistency of these inputs, BRPBFT computes a trust score for each participant, reflecting their perceived trustworthiness within the network. The BRS) is defined by the following equation [16]:

$${\text{Beta Reputation Score}}_{i}=\frac{\alpha_{i}}{\alpha_{i}+\beta_{i}}$$ (1)

where:

- $\alpha_{i}$ and $\beta_{i}$ are the number of positive contributions and negative contributions for user *i*, respectively.

Eigen Trust leverages concepts from graph theory and network analysis to evaluate trust relationships among participants in a decentralized system [17]. In Eigen Trust, each participant assigns trust values to their peers based on past interactions and feedback from other participants. These trust values are then aggregated and propagated iteratively across the network using eigenvector centrality calculations. Through this iterative process, Eigen Trust generates a global trust ranking that reflects the overall reputation of each participant in the network. The calculation considers factors such as the number of connections, the quality of interactions, and the reputation of neighboring nodes, resulting in a more comprehensive assessment of trustworthiness. The Eigen trust algorithm is defined by the following equation:

r(t+1)=T·r(t) (2)

where:

- *r(t+1)* is the trust vector at iteration *t + 1*,
- *T* is the trust matrix, with *Tij* representing the trust that peer *i* has in peer *j, r(t)* is the trust vector at iteration *t*

The trust matrix in the context of Eigen trust is a representation of the trust relationships between peers in a network. It is not necessarily the same as the adjacency matrix, although it can be derived from it depending on the specific implementation. In a trust matrix, each entry *Tij* represents the trust that peer *i* has in peer *j*. This trust can be computed based on various factors such as direct interactions, feedback, or endorsements between peers. The values in the trust matrix typically range between 0 and 1, where 0 indicates no trust and 1 indicates full trust. While the adjacency matrix represents connections between nodes in a network, the trust matrix focuses specifically on trust relationships, which may not always align with the connections themselves. Therefore, while the trust matrix may be derived from the adjacency matrix in some cases, it is a separate concept that emphasizes trust rather than mere connectivity.

In terms of calculation complexity, the Beta Reputation Score is relatively straightforward, involving probabilistic models and Bayesian inference techniques. It evaluates the credibility and consistency of feedback to generate trust scores for participants. Eigen Trust, on the other hand, requires more computational resources due to its iterative nature and the calculation of eigenvector centrality. It considers network topology and connectivity, as well as the reputation of neighboring nodes, to compute trust rankings, which are analysed in the following section.

VRF, is a function that takes an input and generates a pseudo-random output along with a proof that the output was correctly derived, which can also introduce fairness and unpredictability in node selection, mitigating bias. By integrating these elements, BR-PBFT improves security, efficiency, and decentralization, making it a more suitable consensus model for healthcare blockchain systems. VRF, which introduces cryptographic randomness to maintain verifiability is defined as:

Each node *i* generates a VRF output *y*_*i*_ using its private key *ski*:

$$VRF_{sk_{i}}(m)=(y_{i},\pi_{i})$$ (3)

where:

- *y*_*i*_ is the pseudo-random output.
- $\pi_{i}$ is the proof that *y*_*i*_ was correctly computed.

The proof $\pi_{i}$ is broadcasted to all nodes, allowing them to verify that the leader selection process was conducted fairly. A node is selected as the leader based on a combination of its VRF output and its Beta Reputation Score:

$$Leader=\arg\max_{i}\left(w_{1}\cdot {\text{BetaRep}}_{i}+w_{2}\cdot y_{i}\right)$$ (4)

where:

- ${\text{BetaRep}}_{i}$ is the Beta Reputation Score of node *i*.
- *y*_*i*_ is the VRF output.
- *w*_1_ and *w*_2_ are weights balancing reputation and randomness.

By integrating VRF with a reputation-based selection, BR-PBFT ensures a leader election process that is both trust-aware and unbiased, mitigating the risks of malicious collusion and centralization.

### 1.2 Motivation

This research is motivated by the need for a trust-enhanced, scalable, and energy-efficient consensus mechanism tailored for healthcare applications. In traditional PBFT implementations, node selection is arbitrary, leading to potential bias and security risks. Reputation-based models, such as the Beta Reputation Score, provide a mechanism for evaluating node trustworthiness, ensuring that only reliable participants contribute to the consensus process. Additionally, integrating VRF introduces a fairness factor, reducing the risk of centralization and ensuring unbiased leader selection.

To address these concerns, this study introduces BR-PBFT, a novel PBFT variant incorporating a reputation-based node selection mechanism and VRF to enhance trust, efficiency, and fairness in healthcare blockchain applications. This paper provides a comprehensive evaluation of BR-PBFT, highlighting its advantages over traditional PBFT models in terms of computational overhead, scalability, and security.

This research investigates consensus algorithms that achieve the specific requirements of healthcare institutions. The exploration includes basic PBFT, variants of PBFT and PoW approaches to achieving consensus in distributed networks. On the other hand, PoW, renowned for its robustness and security, relies on solving complex cryptographic puzzles to validate transactions. While PoW offers a high degree of security, its significant energy consumption and slower transaction processing times pose challenges for healthcare applications where efficiency and low latency are essential.

By adopting these optimized Byzantine consensus algorithms, healthcare blockchains can achieve high throughput and low latency, crucial for managing large volumes of sensitive medical data. In addition, these algorithms ensure robust security and fault tolerance, maintaining data integrity and confidentiality. As a result, healthcare blockchains can operate more efficiently, providing secure and scalable solutions for data sharing, interoperability, and regulatory compliance. This shift towards more efficient consensus mechanisms represents a significant advancement in the application of blockchain technology in healthcare, addressing the unique demands of the industry while overcoming the limitations of traditional consensus algorithms.

The rest of the paper is organized as follows: Related Works on the consensus mechanisms and their application is presented in Sect 2, followed by the Problem statement in Sect 3. Sect 4 is the proposed methodology consist of analysis of current consenus mechanisms and the novel method -BRPBFT. Sect 5 presents the performance evaluation of the proposed methodology. Sect 6 presented a discussion of the findings and future scope of the enhanced healthcare practices with integrated consensus blockchain, followed by the conclusion, respectively.

## 2 Related works

Blockchain technology has significant potential to transform various industries by improving data management, security, and operational efficiency by chain tracking, access control, and digital identity verification [18]. Decentralization is a pivotal feature in blockchains, crucial for thwarting malicious assaults like the 51 % attack and takeover attempts [19,20]. It has found applications across various sectors, driving innovation in security, trust, and efficiency. In Machine Learning (ML), blockchain ensures data integrity and secure model sharing, enabling federated learning without exposing raw data [21]. These diverse applications highlight blockchain’s potential in enhancing security, decentralization, and operational trust across multiple domains [22]. In the cryptocurrency space, the main focus is on achieving decentralization, security, and resistance through consensus.Therefore, consensus is considered as the backbone of the blockchain, which can also be called blockchain protocol.

Traditional consensus algorithms, such as PoW and PoS, are widely used in the cryptocurrency sector to ensure security and decentralization [23]. However, these algorithms face significant challenges, particularly high computational costs and energy consumption. PoW, for instance, requires participants to solve complex cryptographic puzzles, leading to substantial electricity usage and hardware requirements [24]. In [25] Panda *et al*. provide a comprehensive examination of the industry’s growth, highlighting the protocols and challenges faced by stakeholders. Ibañez *et al*. [26] focus on the significant environmental concerns associated with Bitcoin, particularly its high energy consumption and resulting ecological footprint.

Blockchain technology is being increasingly adopted across various industries, along with finance industries for secure transactions, and healthcare for managing medical records [27]. The integration of this technology into the healthcare industry is increasingly addressing various challenges and improving outcomes [28].

In [29] Rehman *et al*. delve into the trust dynamics within blockchain and cryptocurrency environments. Their research emphasizes the critical role of trust in fostering a secure and efficient cryptocurrency ecosystem, addressing both technological and managerial aspects. Interoperability, consent management, scalability, and regulatory considerations are some challenges that need to be addressed for successful implementation of blockchain technology in healthcare settings [30].

To address these challenges, especially in the context of healthcare blockchains, research has introduced optimized Byzantine consensus algorithms [31]. These algorithms, such as PBFT and variations thereof, are specifically designed to enhance scalability and reduce network overhead [32]. PBFT operates efficiently in environments with known and trusted nodes, making it ideal for permissioned blockchain networks typical in healthcare settings. In the blockchain, trust is established among parties through a distributed network, a non-tamperable cryptographic ledger, and consensus algorithms, with many researchers improving these algorithms for better performance and applying optimized or combined consensus mechanisms for specific use cases [33].

In [34] a Double-Layer Byzantine Fault Tolerance (DLBFT) has been introduced with a hierarchical network structure, reducing message exchanges by 84% and consensus time by 70%, enhancing scalability for Building Information Modeling (BIM) exchanges. A novel PBFT protocol with repairable voting nodes enhances performance and reliability through a multi-dimensional Markov process, offering insights into throughput, availability, and reliability in blockchain systems [32]. Furthermore, the BW-PBFT algorithm improves node quality through credit-based selection, mitigating network bandwidth occupation and enhancing consensus efficiency. These advancements collectively contribute to making PBFT and its derivatives more scalable and efficient, reducing communication overhead.

PoW and PoS [35] are considered to be widely used in cryptocurrency and, on the other hand, in healthcare applications, which require more specialized consensus mechanisms, such as PBFT and Proof of Authority (PoA). The consensus mechanisms used in both domains focus on their design, advantages, and limitations. Furthermore, the integration of PBFT in lightweight blockchain algorithms for healthcare applications ensures security and efficiency by integrating highly trusted nodes and advanced selection methods [36]. These advancements in consensus mechanisms not only enhance the security and integrity of healthcare data but also improve the overall efficiency and reliability of blockchain systems in the healthcare industry [37].

Consensus mechanisms in healthcare blockchain are vital for addressing energy consumption, trust, and bias challenges. Hybrid models enhance trust by assigning reputation based on block activities, reducing CPU and memory usage. Trust-based consensus methods further improve efficiency by quantifying trust, selecting representative nodes, and preventing centralization. These approaches ensure secure, energy-efficient, and fair blockchain systems. A comparative analysis of consensus algorithms is essential to identify the most suitable model for specific healthcare applications, optimizing blockchain adoption in the sector.

## 3 Problem statement

The integration of blockchain in healthcare is driven by the need for secure, efficient, and trust-based data exchange. However, existing consensus mechanisms present significant challenges that limit their applicability in real-world healthcare scenarios. These challenges include:

- High Computational Overhead: Traditional mechanisms like PoW and PBFT require significant computational resources due to their extensive communication rounds and verification processes. This leads to high latency and energy consumption, making them inefficient for real-time healthcare applications that require rapid transaction finalization.
- Lack of Trust Management: PBFT and its variants lack an integrated reputation-based mechanism, making them susceptible to malicious nodes and Sybil attacks. Without a robust trust model, Byzantine nodes can manipulate the network, reducing reliability and security in critical healthcare data exchanges.
- Scalability Limitations: As healthcare systems generate an increasing volume of patient records and transactions, existing consensus mechanisms struggle to efficiently handle network growth. High communication complexity and resource-intensive validation procedures hinder scalability, leading to delays and bottlenecks.
- Security Vulnerabilities: Byzantine nodes can compromise data integrity and network trustworthiness. The absence of a dynamic leader selection mechanism increases the risk of centralization and malicious leader takeovers, potentially jeopardizing patient data security.

To overcome these limitations, this study proposes BR-PBFT, which integrates BRS and VRF to enhance trust, fairness, and efficiency in blockchain-based healthcare systems. By ensuring that, only high-reputation nodes participate in the consensus mechanism, BR-PBFT significantly improves security while maintaining decentralization and computational efficiency, making it a viable solution for real-world healthcare applications.

## 4 Proposed methodology

**Fig 1:**
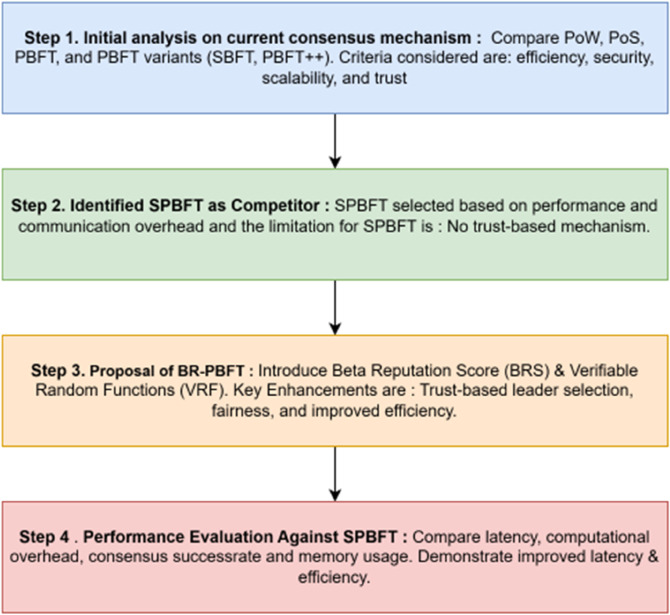
Flow chart of the proposed methodology.

In this section, we outline the methodology following an analysis of different consensus mechanisms to propose a state-of-the-art method, the BR-PBFT consensus, which demonstrates improved computational complexity and reduced latency compared to the existing consensus mechanisms in the healthcare blockchain. The Fig 1 shows the flow of the methodology adopted in this paper. It begins with an initial analysis of various consensus mechanisms, including PoW, PoS, PBFT, and PBFT variants (such as SBFT, PBFT++, and Honey Badger), based on criteria like efficiency, resource utilization, stability, and scalability. For evaluation scope, we focus on BFT baselines given our requirement for deterministic finality and tolerance of misbehavior among admitted nodes. From this analysis, SBFT is identified as the primary competitor due to its superior performance and lower communication overhead, though it lacks a trust-based mechanism. To address this gap, BR-PBFT is proposed, incorporating BRS and VRF to enhance trust-based leader selection, fairness, and efficiency. Finally, BR-PBFT is evaluated against SBFT, demonstrating improvements in latency, computational overhead, consensus success rate, and overall security and efficiency.

**Fig 2:**
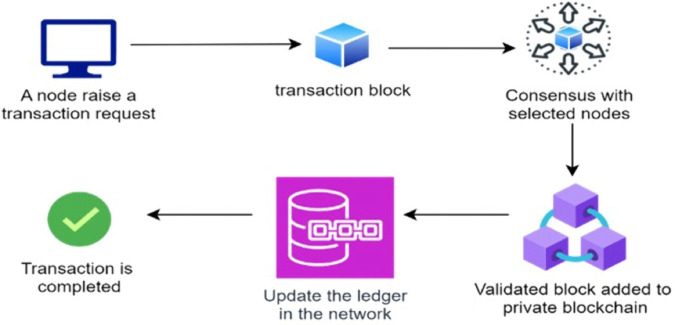
States in a transaction flow.

As consensus is a process of agreeing on a new transaction, depending on its validity, the different states of the healthcare blockchain system, focusing on the workflow of the transactions with consensus involved, are shown in Fig 2. A transaction in a healthcare blockchain refers to any exchange of information or assets between entities within the healthcare system, recorded on the blockchain to ensure transparency, security, and immutability. From the initiation of a transaction by a user to its validation by consensus nodes and eventual recording in the distributed ledger, each phase plays a crucial role in upholding the reliability and security of the healthcare blockchain ecosystem.

Here transactions begin when a user initiates a request, such as uploading or viewing a medical record. The client application formulates the transaction with details like sender, receiver, and data, then broadcasts it to the network. Upon receipt, distributed nodes perform initial validations to ensure protocol compliance before forwarding it to consensus nodes. The consensus mechanism then verifies the transaction’s legitimacy, ensuring network-wide agreement. Once validated, the transaction is permanently recorded in the blockchain ledger, grouped into a block, and appended to the chain. This update ensures that all nodes maintain a synchronized, secure, and tamper-resistant record. Some of the expected challenges in the system are discussed below.

- **Expected Challenges**: Cryptocurrency systems rely on computationally intensive models to ensure transaction consensus and enhance security, often prioritizing these features over immediate data access. In contrast, healthcare systems require rapid access to medical records, especially during emergencies, where delays could compromise patient care. However, in blockchain-based healthcare systems, consensus mechanisms primarily affect write operations rather than read operations. This means that critical patient data can still be accessed promptly during emergencies, as read operations do not require consensus. For write operations, transactions can be securely queued and processed once the emergency has passed, ensuring both data integrity and timely care. Another challenge is the energy-intensive nature of certain blockchain consensus mechanisms, which may attract regulatory scrutiny due to environmental concerns. While mining-based mechanisms, like Proof-of-Work, are particularly resource-heavy, alternative methods such as PBFT and its variants are more energy-efficient and better suited to healthcare’s 24/7 operational model.
- Maintaining the integrity and security of the healthcare blockchain also presents unique challenges. A malicious entity within the system could exploit their access to sensitive patient data. For instance, a node posing as a legitimate doctor might aim to steal or misuse patient information. This risk is exacerbated when the same set of nodes consistently participates in the consensus process, potentially leading to biases and reduced system integrity. To mitigate this, the healthcare blockchain must incorporate robust mechanisms to prevent repeated involvement of identical nodes, such as random node selection and reputation-based participation criteria. These measures ensure that consensus decisions are unbiased and that all participating nodes remain trustworthy and reliable.

### 4.1 Design goals

Based on the above discussion, the following design goals can be considered:

- Response time: Given the critical nature of healthcare data and the need for immediate access to medical records, the blockchain system must prioritize rapid response times. Any delay in data retrieval could adversely affect patient care, particularly in emergencies. Therefore, the design must ensure that the system can handle high-frequency access requests efficiently, providing near-instantaneous data retrieval.
- Energy consumption: The energy-intensive nature of traditional blockchain consensus mechanisms, such as proof-of-work, is impractical for healthcare applications. The system should aim to minimize energy consumption to ensure sustainability and compliance with environmental regulations.
- Trust: Maintaining the integrity and security of the blockchain system is paramount, especially in a sector as sensitive as healthcare. The system must include robust measures to ensure that all participating nodes are trustworthy.
- Bias: To prevent potential biases and ensure fair participation in the consensus process, the system must implement mechanisms to diversify node involvement. The goal is to prevent any single node or group of nodes from exerting undue influence over the network, thereby maintaining the overall trustworthiness and impartiality of the system.

### 4.2 Analysis and discussion on current design and methods

The parameters discussed in the preceding sections were carefully considered during the analysis. Write transactions in blockchain systems are documented as part of a block on the ledger, ensuring the immutability and traceability of data. This characteristic emphasizes the importance of investigating the effects of increasing node or block growth on emerging blockchain technologies and algorithms. Identifying optimal consensus mechanisms is critical to balancing scalability, performance, and security in healthcare systems.

Consensus is a fundamental component of blockchain operations. In the context of digital currencies, it ensures data integrity and prevents fraudulent activities. This study examines well-known algorithms like PBFT and Proof-of-Work (PoW) while proposing enhancements tailored to the unique needs of the healthcare industry, where rapid, secure, and efficient data handling is paramount.

The PBFT, BR-PBFT, and SPBFT consensus algorithms were implemented in Python, utilizing the *pycryptodome* library for RSA key generation, message signing, and verification. Performance metrics such as CPU and memory usage were tracked with the *psutil* library, while Python’s *concurrent.futures* module facilitated multi-threaded operations for simulating prepare and commit phases. Visualization of results, including resource usage and latency comparisons across varying node counts, was achieved using the *matplotlib* library.

#### 4.2.1 Analysis of PoW vs PBFT.

**Fig 3:**
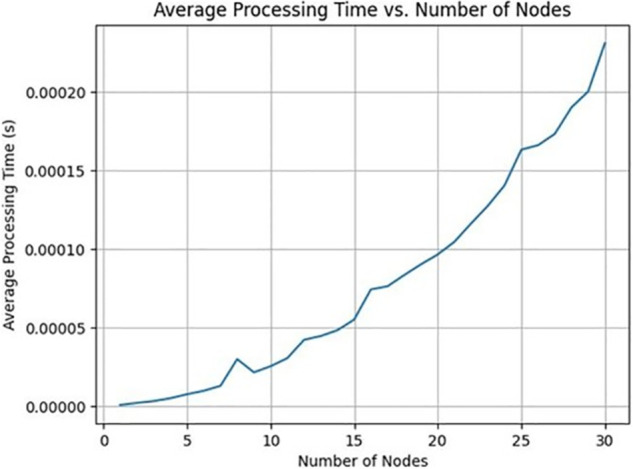
Impact of node count on average processing time in PBFT consensus.

In the PBFT consensus algorithm, each node in the network maintains a list of other nodes and communicates with them to achieve consensus on the validity of transactions. As the number of nodes added to the blockchain increases, the processing time in PBFT indeed escalates, as shown in Fig 3. This escalation occurs due to the expansion of the network’s size, leading to an increase in the number of messages exchanged and the complexity of communication among nodes. However, this can be managed by introducing off-chain storage such as the Interplanetary File System (IPFS) [38]. IPFS is a decentralized storage network that aims to make the web faster, safer, and more open. By introducing off-chain storage solutions like IPFS, blockchain systems can alleviate some of the processing burdens associated with storing large amounts of data directly on the blockchain. With IPFS, data is stored and retrieved based on content rather than location, making it more scalable and efficient for storing large files or data sets. Offloading non-essential data or large files to IPFS can reduce the processing overhead on the blockchain nodes, thereby mitigating the impact of increased processing time due to the addition of more nodes.

While it is inferred from the literature review that PoW governs the cryptocurrencies on blockchain platforms, the stability and resource consumption comparison of PBFT and PoW sheds much-needed light on the demands of both fields. Stability in consensus is essential for the overall health and functionality of a decentralized network, as it ensures that the network can continue to operate effectively even in the face of various challenges.

**Fig 4:**
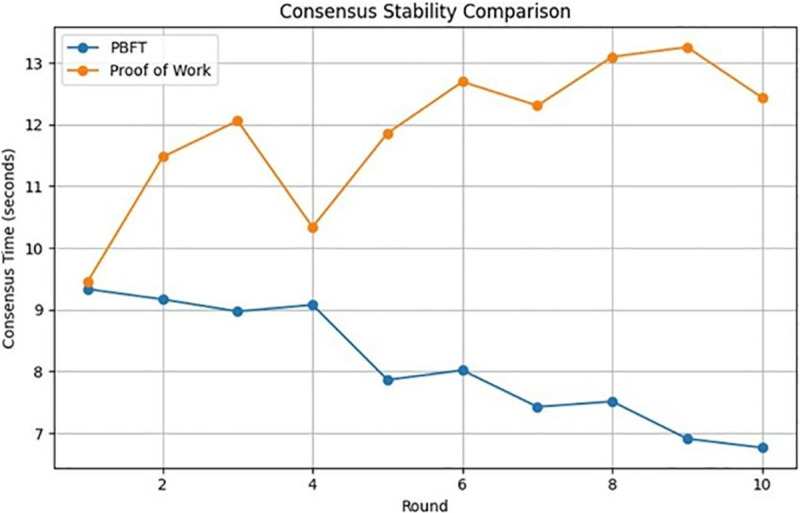
PoW vs PBFT based on consensus stability.

**Fig 5:**
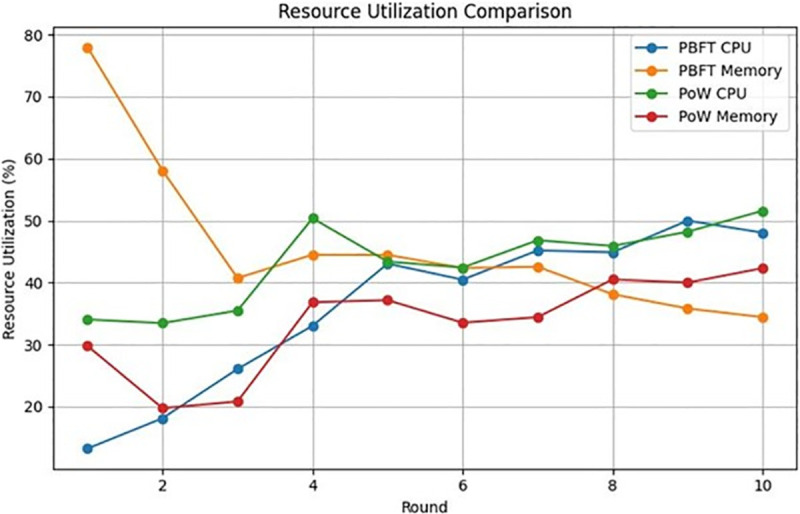
PoW vs PBFT based on resource utilization.

Fig 4 illustrates the stability of the consensus, defined as the time it takes for the nodes to agree on a block during the consensus process. This metric provides insights into the efficiency and reliability of the consensus algorithm under various network configurations. From Figs 4 and 5, using PoW for a healthcare use case can be an exhaustive task, compromising consensus stability as well as resource consumption level. This is particularly due to the complex mining procedure involved in PoW, which can be time-consuming and overly sophisticated for a health care institute. The analysis reveals that PBFT is generally faster and more scalable compared to PoW. It can achieve consensus with lower latency, making it suitable for applications that require fast transaction confirmation times, such as healthcare where quick access to patient data may be critical. PoW consensus can suffer from scalability issues as the network grows, leading to longer confirmation times and higher transaction fees during periods of network congestion. The energy-intensive nature of PoW mining processes, coupled with its significant drain on computational resources, has increasingly drawn regulatory scrutiny due to its environmental impact. Regulatory bodies are becoming more attentive to the ecological implications of PoW, posing potential challenges for healthcare organizations striving to adhere to regulatory standards amidst PoW’s high energy consumption. This heightened scrutiny underscores the need for alternative consensus mechanisms that offer greater efficiency and sustainability.

In contrast, PBFT enjoys a more favourable regulatory perception due to its efficient consensus mechanism. In jurisdictions with stringent energy conservation policies, PBFT is often viewed as a sustainable alternative to PoW. Additionally, PBFT’s reliance on a predefined set of nodes for consensus aligns well with healthcare institutions’ sustainability objectives. By opting for PBFT over PoW in blockchain applications, healthcare organizations can ensure regulatory compliance while contributing to environmentally responsible practices. The efficiency of PBFT further enhances its appeal for healthcare entities navigating regulatory requirements and sustainability concerns, making it an attractive option for adoption within the sector.

#### 4.2.2 Analysis of the variants of PBFT.

A detailed review of current PBFT variants is essential before developing a new and improved version of PBFT. By identifying the implementations’ advantages, disadvantages, and gaps, this analysis will lay the groundwork for developing a consensus method that is both more secure and effective. The presence of Byzantine nodes in a distributed system are those that may deviate from the prescribed protocol or behave maliciously. These nodes can disrupt consensus by sending conflicting messages or misleading other nodes. Byzantine fault tolerance mechanisms are designed to withstand such behaviour, ensuring the integrity and reliability of the network.

**Fig 6:**
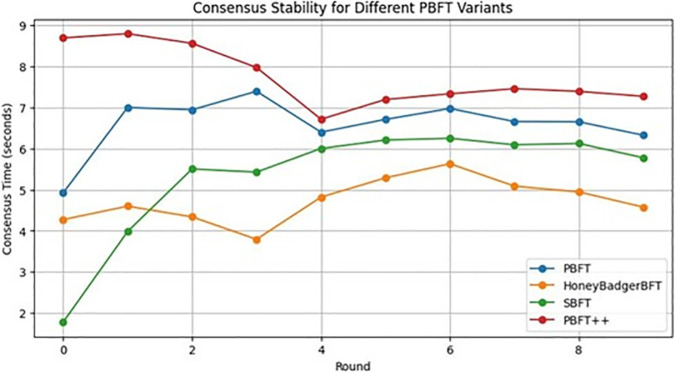
PBFT variants without byzantine nodes.

**Fig 7:**
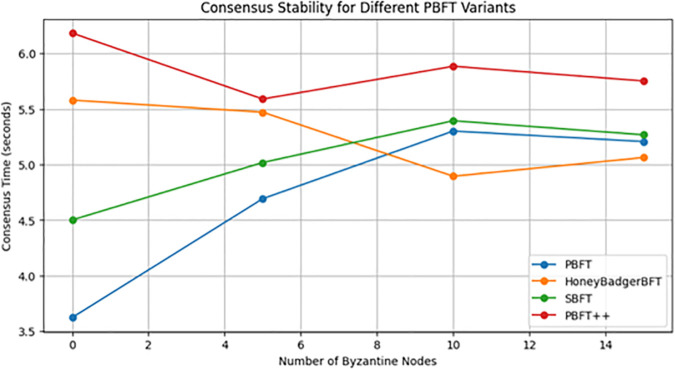
PBFT variants with byzantine nodes.

When evaluating the consensus stability as shown in Figs 6 and 7 based on the number of rounds and presence of Byzantine nodes on four PBFT variants—Traditional PBFT, PBFT++, Honey Badger BFT, and SBFT—in the realm of healthcare blockchain applications, distinct traits and cryptographic protocols emerge. Each round represents a complete execution of the consensus process (pre-prepare, prepare, commit) but does not include appending transactions to a blockchain ledger. The simulation focuses on evaluating the computational and communication performance of the consensus algorithms. Traditional PBFT offers Byzantine fault tolerance, with consensus times initially moderate amid growing Byzantine nodes, eventually stabilizing as the network adapts to healthcare data integrity demands. PBFT++ introduces optimizations, potentially lengthening initial consensus times due to added complexity yet ensuring stability over time, critical for safeguarding the security and integrity of patient records in healthcare blockchains.

Honey Badger BFT may initially elevate consensus times, referring to potential overhead during transient phases, such as system setup or network reconfiguration. This is due to the asynchronous nature of the protocol, which accommodates random message delays and additional cryptographic operations. However, as shown in Fig 7, Honey Badger BFT demonstrates lower steady-state consensus times than PBFT, highlighting its efficiency. Here, the term reinforcing robustness refers to the protocol’s ability to tolerate ⌊n/3⌋ faulty nodes, maintain liveness and safety in asynchronous environments, and ensure reliable performance under varying network conditions.

Therefore, initially, this may increase consensus times as the system adjusts to its asynchronous nature, but ultimately reinforces robustness in healthcare data sharing and access control. SBFT streamlines PBFT for improved efficiency and scalability, maintaining moderate initial consensus times and achieving stability over time, thereby suiting environments processing high volumes of healthcare transactions securely.

The selection of a PBFT variant for a healthcare institute’s blockchain implementation requires careful consideration of performance, security, and compliance with healthcare data regulations. Traditional PBFT, with its established protocols and reliability, fits institutes prioritizing stability. PBFT++, balancing security with scalability, suits organizations handling large healthcare data volumes. Honey Badger BFT, although initially slower due to decentralization, enhances data integrity, making it suitable for robust healthcare data sharing. SBFT’s efficiency and scalability make it ideal for high-volume transaction processing. Because SBFT’s pipelined consensus mechanism, optimized communication complexity, and dynamic quorum selection make it particularly suitable for environments processing high volumes of healthcare transactions securely. While Honey Badger BFT and PBFT demonstrate lower consensus times in some scenarios, their designs face scalability challenges under heavy transaction loads, making SBFT better equipped for high-throughput applications. In addition, regulatory compliance, network size, transaction throughput, and resource availability must be evaluated to align with the institute’s long-term objectives in the safe management of healthcare data within a decentralized network.

Based on the analysis of many variants, augmenting some trust metrics with PBFT can be a viable strategy to increase the effectiveness, security, and dependability of the consensus process. By using a reputation-based trust score, the system can dynamically modify trust levels in response to nodes’ behavior and performance, which lessens the effect of unreliable or malevolent players. This improvement not only improves node selection but also lowers network latency overall and boosts network robustness, strengthening the consensus mechanism’s resistance to failures and attacks. Therefore, incorporating a reputation score mechanism can significantly enhance the consensus process. A new variant that leverages reputation scores can prioritize nodes with higher reliability and performance, thereby reducing the likelihood of selecting malicious or faulty nodes as primaries. This approach not only improves the overall security and fault tolerance of the network but also enhances efficiency by potentially lowering communication overhead and consensus latency. By dynamically adjusting to the behavior of nodes over time, a reputation-based PBFT variant can maintain robust performance even under adverse conditions, making it a compelling solution for critical applications like healthcare and finance, where security and reliability are paramount.

#### 4.2.3 Analysis of reputation score.

**Fig 8:**
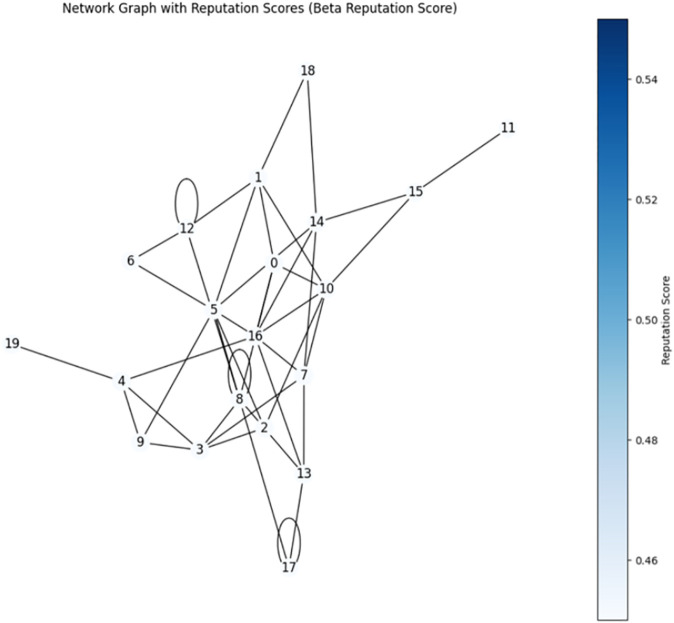
Network graph of beta reputation score.

**Fig 9:**
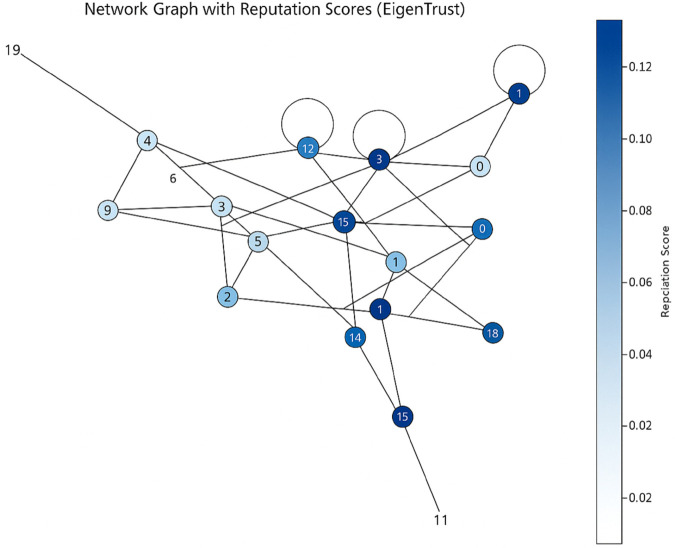
Network graph of eigen trust score.

A reputation score helps establish the credibility of individuals and entities by assigning a numerical value to participants, fostering a more secure and trustworthy environment. Here, in the analysis of the Beta Reputation Score and Eigen Trust Score, a network graph has been utilized. The network graph depicted on Figs 8 and 9 provides a visual representation of the blockchain network, where nodes are depicted as circles (or vertices) and interactions between nodes are depicted as edges (or lines). This graphical representation enables stakeholders to easily comprehend the structure and dynamics of the network. Each node represents an entity within the blockchain, such as a healthcare provider, patient, or data repository. Interactions between these nodes, such as data exchanges, transactions, or communications, are illustrated by the connecting edges.

A key feature of this visualization is the color-coding of nodes based on their reputation scores. Reputation scores are visually encoded using a color gradient. Nodes with higher reputation scores are assigned colors that typically represent higher trust levels (e.g. blue), while nodes with lower reputation scores are assigned colors indicating lower trust levels (e.g., pale blue). A color bar or legend is provided to indicate the range of reputation scores and their corresponding colors, allowing viewers to quickly gauge the trustworthiness of different nodes within the network.

This visualization serves multiple purposes. Firstly, it aids in identifying highly trusted nodes that can be relied upon for secure and accurate data transactions. Secondly, it helps identify potentially untrustworthy or malicious nodes that can pose risks to the integrity of the network.

By observing the network graphs generated using the Beta Reputation Score and Eigen Trust algorithms, users can visually assess how reputation scores are distributed across nodes and how different nodes interact with each other.

Choosing between Eigen Trust and Beta Reputation Score for a healthcare blockchain use case depends on several factors, including the nature of interactions between participants, and considerations regarding privacy and scalability. Based on this analysis, the reputation scores used can alter depending on the hierarchical structure of the healthcare institution, which is summarized in Table 1.

**Table 1:** Reputation scores suitable for different healthcare settings based on analysis.

Trust Score	Reasoning
Eigen Trust	Small teams with close interactions. Suitable for direct interaction-based trust assessment. Examples: Primary care clinics, Specialized Clinics
Beta Reputation Score	**Case - I** Large, complex organizations. Easier reputation evaluation for departments and individuals. Example: Hospitals
	**Case - II** Encompasses multiple facilities. Structured evaluation based on collaborations. Example: Healthcare Networks/Systems
	**Case - III** Coordination among networks, agencies, and stakeholders. Evaluation at the regional/national level based on contributions. Example: Regional/National Health Systems

### 4.3 A reputation integrated consensus model for blockchain-based healthcare systems: BR PBFT

**Fig 10:**
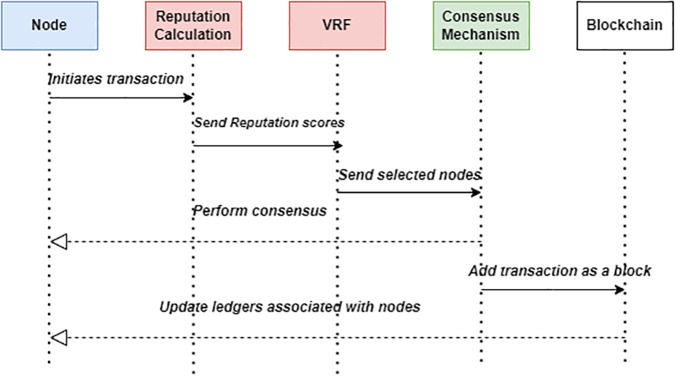
Workflow diagram of the proposed solution.

From the analysis, incorporating reputation scores into the PBFT consensus mechanism, alongside VRF, presents a highly effective approach for enhancing security and efficiency in healthcare blockchain networks. In the system, reputation scores can be used to ensure that only highly reputable nodes participate in the consensus process, significantly reducing the likelihood of malicious activities and improving the overall trustworthiness of the network. This selective participation is crucial in healthcare, where data integrity and confidentiality are paramount. VRFs generate random values that can be publicly verified, ensuring that the selection process cannot be manipulated. This combination of reputation scores and VRFs improves the decentralization and resilience of the blockchain network by ensuring that nodes are chosen based on both their reliability and a fair random process. The workflow of the proposed solution is shown in Fig 10.

Let *i* be the node whose reputation is being updated in round *t*, and let ${ℛ}_{t}(i)$ be the set of authenticated raters that issued feedback for *i* in round *t*. Each rater $r\in {ℛ}_{t}(i)$ provides *binary* feedback $\left(f_{r\to i}^{+},f_{r\to i}^{-})\in \{0,1\right\}$. Maintain Beta parameters $\left(\alpha_{i},\beta_{i}\right)$ for node *i*.


$$\alpha_{i}^{\prime }\;=\;\alpha_{i}\;+\;\sum_{r\in {ℛ}_{t}(i)}\gamma_{r}\;f_{r\to i}^{+},\;\beta_{i}^{\prime }\;=\;\beta_{i}\;+\;\sum_{r\in {ℛ}_{t}(i)}\gamma_{r}\;f_{r\to i}^{-},$$

$${BetaRep}_{i}(t)\;=\;\frac{\alpha_{i}^{\prime }}{\alpha_{i}^{\prime }+\beta_{i}^{\prime }}.$$

**Rater credibility.** Let $R_{r}(t-1)\in [0,1]$ denote rater *r*’s Beta reputation from the previous round (use 0.5 for new raters) the credibility weight can be calculated as:


$$\gamma_{r}\;=\;\max\;(\gamma_{\min},\;R_{r}(t-1{)}^{\eta }),\;\eta \in (0,1],\;\;\gamma_{\min}\in [0,0.2].$$

*R*_*r*_(*t*−1) is rater *r*’s previous-round Beta reputation in [0,1]; use *R*_*r*_(*t*−1) = 0.5 (neutral) if the rater is new. The sharpening exponent *η* satisfies η≥1 (e.g., 2); larger *η* penalizes low-reputation raters more. The credibility floor $\gamma_{\min}$ (e.g., 0.1) ensures no rater’s weight becomes exactly 0. The credibility exponent *η* (e.g., 0.5) softly compresses extremes so neither very low nor very high prior reputations dominate a single round, while the credibility floor $\gamma_{\min}$ (e.g., 0.1) prevents zero influence and caps low-trust raters, mitigating collusion. All feedback is digitally signed and accepted only from authenticated participants in the permissioned network, ensuring accountability.

**Leader election with verifiable randomness (VRF).** To avoid predictable or repeatedly biased leader choices, BR-PBFT augments reputation with a VRF. For each eligible validator *i*, compute


$$(y_{i},\;\pi_{i})\;=\;{VRF}_{sk_{i}}(m_{t}),$$

where $y_{i}\in [0,1]$ is a pseudorandom output and $\pi_{i}$ is a non-interactive proof of correctness. Peers verify the proof before proceeding:


$$Verify(pk_{i},\;m_{t},\;y_{i},\;\pi_{i})\;=\;\text{true}.$$

We combine trust and randomness via


$$s_{i}\;=\;w_{1}\cdot {BetaRep}_{i}\;+\;w_{2}\cdot y_{i},\;w_{1},w_{2}\in [0,1],\;w_{1}+w_{2}=1,$$

and select the primary as $\arg\max_{i}s_{i}$ among the eligible validators. Here *w*_1_ and *w*_2_ control the balance between reputation and randomness (e.g., $w_{1}\;=\;0.8$, $w_{2}\;=0\;\mathrm{.2}$), preserving the benefits of trust while ensuring unpredictability. Each candidate generates one proof $\left(y_{i},\pi_{i}\right)$ and peers perform one verification; this adds negligible cost relative to PBFT’s *O*(*n*^2^) message exchanges. A missing/invalid $\pi_{i}$ disqualifies the candidate; the next-highest *s*_*i*_ is chosen deterministically, ensuring progress without weakening PBFT quorums. Algorithm 1 illustrates the process of BR-PBFT, while Algorithm 2 defines the process of finding the credibility-weighted Beta Reputation.

In the final phase, the selected nodes perform consensus on the initiated transaction. This involves verifying the transaction details and agreeing on their validity. Once consensus is reached, the consensus mechanism updates the blockchain by adding the new transaction as a block. Additionally, the ledgers associated with the participating nodes are updated to reflect their involvement in the consensus process. This entire workflow ensures that transactions are validated in a secure, reliable, and efficient manner, leveraging reputation scores to enhance the trustworthiness of the blockchain network. PBFT forms the foundation by ensuring fault-tolerant consensus through its Pre-Prepare, Prepare, and Commit phases, enabling reliable validation of transactions even in the presence of faulty or malicious nodes. Therefore, BR-PBFT introduces fairness and randomness in node selection while prioritizing highly reputable nodes, thereby reducing communication overhead and improving scalability.

#### 4.3.1 Security and trust management.

- Collusion resistance: To prevent groups of nodes from mutually inflating each other’s reputation, BR-PBFT weights every rating by the rater’s prior trustworthiness. Ratings from low-trust raters carry only bounded influence and cannot rapidly boost peers unless already-credible participants agree. Feedback is digitally signed, originates only from authenticated participants in the permissioned network, and is rate-limited (e.g., one rating per rater per target per round). We also cap who can influence consensus by requiring a reputation eligibility threshold and then taking a Top-k shortlist; this bounds the attack surface even if a colluding cluster exists.
- Sybil attack prevention: Participation is permissioned (verifiable identity). New nodes are initialized with conservative priors (α=β=1) and neutral bootstrap reputation R(t−1)=0.5, which limits initial influence via $\gamma_{r}$ and requires sustained good behavior to cross the eligibility threshold $T_{\text{elig}}$ and enter the validator shortlist.
- Reputation poisoning mitigation: To avoid old positives masking recent misconduct, we apply recency weighting to the Beta counts before adding round-*t* feedback. Reputation is updated each round with a recency bias (older history gradually matters less), so recent misconduct cannot be hidden behind long-past positives. Inconsistent or dishonest raters see their future influence reduced, and repeated misbehavior can trigger penalties.

**Algorithm 1 BR-PBFT.**

**Inputs:** nodes *N*, transaction *T*, round *t*

**Params:** *k*, $T_{\text{elig}}$ (eligibility threshold)

**procedure** InitiateTransaction(*N*,*T*)

send (*N*,*T*) to ReputationSystem

**end procedure**

**procedure** ReputationSystem(*N*,*T*)

**for** each node i∈N **do**

${\text{BetaRep}}_{i}\leftarrow$ BetaRepCalculation$\left(F_{i},\alpha_{i},\beta_{i},\{\{R_{r}(t-1)\},\eta ,\gamma_{\min}\right)$

**end for**

send $\left\{(i,{\text{BetaRep}}_{i})\right\}$ and *T* to VRFLeaderAndSet

**end procedure**

**procedure** VRFLeaderAndSet($\{(i,{\text{BetaRep}}_{i})\},T$)

${𝒞}_{t}\leftarrow \{i|{\text{BetaRep}}_{i}\geq T_{\text{elig}}\}$

${𝒱}_{t}\leftarrow$ TopK$\left({𝒞}_{t},k\right)$

**for** each $i\in {𝒱}_{t}$ **do**

$\left(y_{i},\pi_{i})\leftarrow {\text{VRF}}_{sk_{i}}(m_{t}\right)$

$s_{i}\leftarrow w_{1}\cdot {\text{BetaRep}}_{i}+w_{2}\cdot y_{i}$

**end for**

$\text{primary}\leftarrow \arg\max_{i\in {𝒱}_{t}}s_{i}$; $\text{replicas}\leftarrow {𝒱}_{t}⧵\{\text{primary}\}$

send $\left(\text{primary},\text{replicas},\{\pi_{i}\},T\right)$ to ConsensusMechanism

**end procedure**

**procedure** ConsensusMechanism($\text{primary},\text{replicas},\{\pi_{i}\},T$)

**Pre-prepare:** primary broadcasts ⟨pre-prepare,T⟩

**Prepare:** replicas verify $\pi_{i}$ and broadcast prepares

**Commit:** on 2f+1 valid prepares, broadcast commits

**if** commit quorum reached **then**

AddTransaction(*T*)

**end if**

**end procedure**

**procedure** AddTransaction(*T*)

append *T* to blockchain; update local ledgers

for each $i\in {𝒱}_{t}$: $\{R_{i}(t)\leftarrow {\text{BetaRep}}_{i}$

**end procedure**

**Algorithm 2 Credibility-weighted beta reputation calculation.**

**Initialization:** α=0.5, β=0.5

**Parameters:** η∈(0,1], $\gamma_{\min}\in [0,0.2]$

**Input:** feedback set *F* = {*f*_*r*_} from raters *r*; each $f_{r}=(f_{r}^{+},f_{r}^{-})\in \{0,1{\}}^{2}$ with $f_{r}^{+}+f_{r}^{-}\in \{0,1\}$; current α,β; prior-round Beta scores {*R*_*r*_(*t*−1)

**Output:** ${\text{BetaRep}}_{i}$

**procedure** BetaRepCalculation

**Compute rater credibility for each r∈F:**

$\gamma_{r}\leftarrow \max\;(\gamma_{\min},\;({\hat{R}}_{r}(t-1)){\;}^{\eta })$

**Update parameters with credibility-weighted feedback:**

$\alpha^{\prime }\leftarrow \alpha +\sum_{r\in F}\gamma_{r}\cdot f_{r}^{+}$

$\beta^{\prime }\leftarrow \beta +\sum_{r\in F}\gamma_{r}\cdot f_{r}^{-}$

**Compute Beta reputation:**

${\text{BetaRep}}_{i}\leftarrow \frac{\alpha^{\prime }}{\alpha^{\prime }+\beta^{\prime }}$

**Persist for next round:**

$\{R_{i}(t)\leftarrow {\text{BetaRep}}_{i}$

**Return** ${\text{BetaRep}}_{i}$

**end procedure**

Collectively, these safeguards strengthen BR-PBFT against collusion, Sybil attacks, and reputation poisoning, addressing common vulnerabilities of reputation systems and ensuring secure and trustworthy consensus in healthcare blockchain environments. The theoretical analysis of the proposed system based on the challenges and design goals discussed above:

- Addressing Response Time: To ensure rapid response times, essential for accessing medical records promptly during emergencies, our system leverages the PBFT consensus algorithm. PBFT is known for its efficiency in achieving consensus with low latency compared to traditional proof-of-work systems. By integrating BetaRep, which selects reliable and efficient nodes based on reputation scores, the system further reduces delays caused by unreliable or slow nodes. This combination ensures that healthcare data can be accessed swiftly, meeting the high-frequency access demands of healthcare environments.
- Optimizing Energy Consumption: Traditional blockchain systems often face criticism for their high energy consumption, particularly those utilizing PoW consensus mechanisms. Our proposed system addresses this concern by employing PBFT, which is significantly less energy intensive as it does not rely on mining processes. Additionally, BetaRep enhances efficiency by ensuring that only the most reliable and well-performing nodes participate in the consensus process, thereby minimizing unnecessary computational efforts. The use of VRF further optimizes resource utilization by introducing fair and efficient node selection, reducing the overall energy footprint of the system.
- Ensuring Trust and Security: Maintaining the integrity and security of healthcare data is paramount. BetaRep plays a crucial role in evaluating the trustworthiness of network participants by aggregating feedback from various sources, such as patient reviews and adherence to regulatory standards. This reputation system ensures that only trustworthy nodes are involved in the consensus process, thereby enhancing the security and reliability of transactions. PBFT complements this by providing a fault-tolerant consensus mechanism that can handle node failures and malicious activities, ensuring the system’s robustness. Together, these components create a secure environment for managing sensitive healthcare data.
- Mitigating Bias in Node Selection: To prevent potential biases and ensure fair participation in the consensus process, the integration of VRF introduces a layer of randomness and fairness to node selection. VRFs generate pseudorandom outputs that others can efficiently verify, ensuring that selecting nodes to participate in PBFT is transparent and tamper-proof. This randomness mitigates the risk of any single node or group of nodes exerting undue influence over the network, maintaining the overall impartiality and trustworthiness of the system.

## 5 Performance evaluation

For the analysis on different variants of PBFT and to evaluate the proposed PBFT variant BR-PBFT with VRF, simulation experiments are adopted node counts ranging from 4 to 20, representing small to medium-sized blockchain networks. Metrics evaluated included latency—the time taken to complete a single round of consensus, measured from the pre-prepare phase to the commit phase—and average CPU and memory usage during each round.

### 5.1 Experimental setup

To ensure the reproducibility of results, the BR-PBFT consensus mechanism was implemented and tested in a controlled simulation environment. The key components of the setup are:

- Hardware Configuration: Experiments were conducted on a system with an Intel Core i7 processor, 16GB RAM, and Ubuntu 20.04 LTS operating system.
- Software Stack: The implementation was developed using Python 3.9, with cryptographic operations handled by the PyCryptodome library and process monitoring using psutil.
- Network Simulation: The consensus algorithm was tested in a simulated blockchain network with 4 to 20 nodes.

The traditional PBFT algorithm was simulated to establish baseline performance metrics, with latency, CPU, and memory usage recorded for each round. BR-PBFT extended the PBFT model by incorporating reputation-based primary selection and fault tolerance, with faulty nodes simulated at a 10% probability of introducing errors during consensus rounds.

SBFT employed a simplified primary selection mechanism for performance comparison, with resource usage and latency measured under the same conditions as PBFT and BR-PBFT. A reputation-based consensus algorithm with VRF for the selection of nodes is analyzed based on latency and resource utilization. Along with these metrics, there are many ways to evaluate the proposed variant, such as throughput, scalability, and fault tolerance.

**Fig 11:**
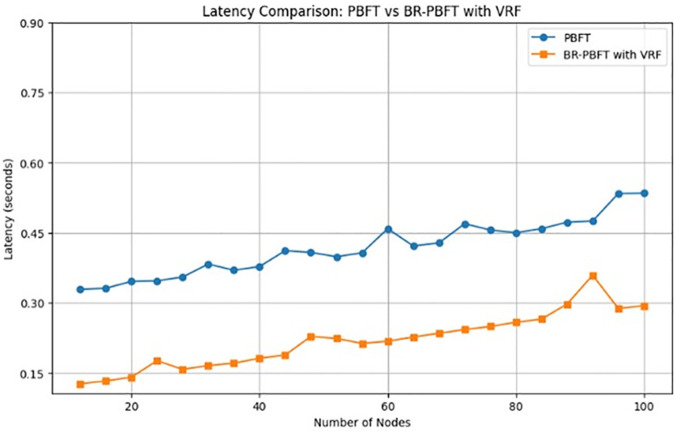
Latency comparison of PBFT and BR-PBFT.

According to the analysis on latency of these two algorithms based on the node count and starting with *α* and *β* set to 1, which makes the initial reputation score calculated by the Beta distribution will be 0.5, representing a neutral or balanced reputation. This allows the reputation to be adjusted based on the outcomes of subsequent transactions, reflecting the node’s behavior over time. The latency for both PBFT and BR-PBFT is recorded across multiple rounds and varying node counts, shown in Fig 11. The results are plotted to compare the performance of the two algorithms in terms of latency.

BR-PBFT algorithm is designed to potentially offer lower latency by using a reputation-based VRF for primary selection and considering only a subset of nodes for consensus. However, the effectiveness of this approach depends on the distribution of reputation scores and the presence of faulty nodes. Nodes with higher reputations are more likely to be selected as primaries, reducing the likelihood of selecting faulty nodes and thereby improving consensus reliability and speed. The simulation considers the impact of faulty nodes, which can disrupt the consensus process. The reputation system helps mitigate this by penalizing faulty behavior.

**Fig 12:**
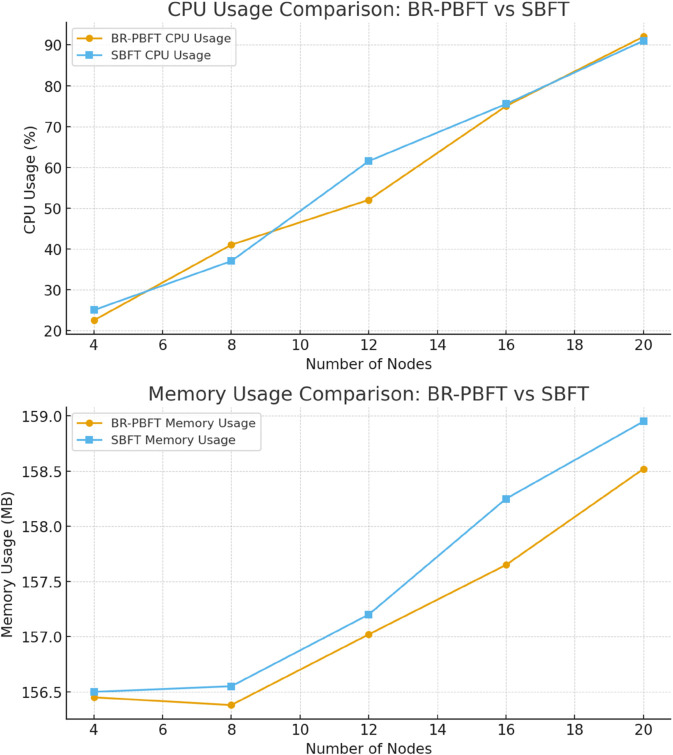
BR-PBFT vs SBFT.

Fig 12 illustrates a comparative analysis of CPU and memory usage between BR-PBFT and SBFT as the number of nodes increases. SBFT is mainly applicable in huge volume transactions, as mentioned earlier. Here in terms of CPU usage, both BR-PBFT and SBFT show a linear increase as the number of nodes scales up. For smaller networks of 4 to 8 nodes, the CPU usage for both protocols remains relatively close. However, as the number of nodes surpasses 10, SBFT begins to exhibit higher CPU consumption, reaching approximately 90% at 20 nodes compared to BR-PBFT’s 80%. Similarly, the memory usage for both protocols also increases linearly with the number of nodes. At smaller network sizes, around 4 nodes, memory usage is nearly identical, with SBFT slightly higher. This trend continues as the network size grows, with SBFT consuming around 159 MB of memory at 20 nodes, while BR-PBFT uses about 158 MB. Overall, BR-PBFT demonstrates more efficient resource utilization, both in terms of CPU and memory, especially as the network size increases, indicating its superior scalability compared to SBFT. In the case of a healthcare environment with consensus and dynamic scalability of network there is a need for an efficient resource utilization which makes BR-PBFT a state-of-the-art light weight solution with better performance.

**Fig 13:**
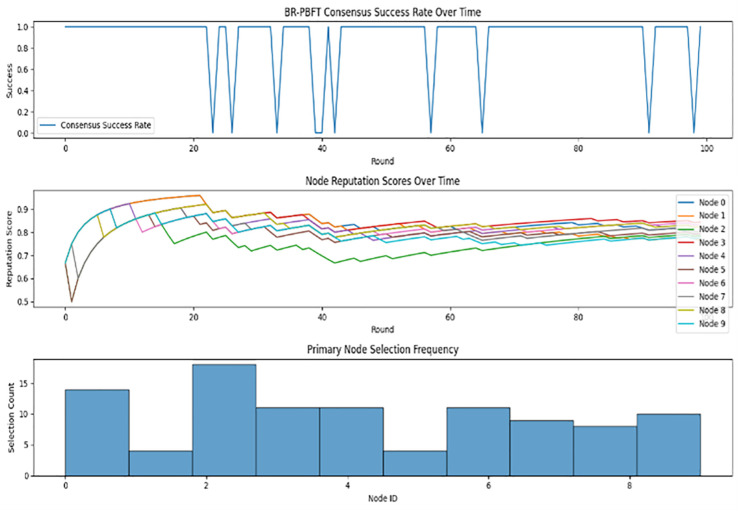
BR-PBFT consensus with VRF.

Based on the system analysis in Fig 13, the high consensus success rate indicates that the BR-PBFT consensus mechanism is robust and capable of achieving agreement among nodes in the presence of faults. The periodic nature of the failures (where consensus drops to zero) might suggest systematic issues or regular occurrences of disruptions that the system is mostly able to recover from in subsequent rounds. The improving reputation scores suggest that the system effectively incentivizes good behaviors and reliable performance from the nodes. Overall, the system appears to be effective in maintaining high consensus success rates and improving node reputations over time.

From the analysis depicted on Fig 13, it is evident that the reputation scores generally improve over time for most nodes, which suggests that nodes are behaving correctly, and their reputation is being positively reinforced. However, there are fluctuations indicating instances where nodes may have been involved in faulty or sub optimal behavior, temporarily reducing their reputation scores before recovering.

**Fig 14:**
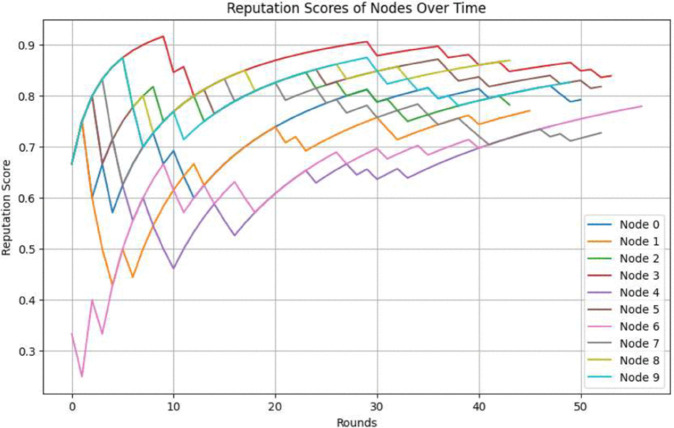
Reputation scores of nodes over time.

Node 0, for instance, starts with a lower reputation score but steadily improves, reaching a high score close to 0.9 by the end of the rounds. In contrast, Node 9 exhibits more variability, indicating it may have had more interactions affecting its reputation both positively and negatively. The overall upward trend for most nodes highlights the effectiveness of the reputation system in differentiating between consistent and inconsistent performers. From Fig 14 over time, it seems that most nodes’ reputation scores tend to stabilize. Here nodes such as Node 0, Node 3, and Node 6 reach relatively high and stable scores around 0.8–0.9, indicating consistent correct behavior. Nodes with initially low scores, such as Node 4 and Node 9, show a slow but steady improvement, although they do not reach as high a stable reputation as others. By analyzing and adjusting these factors, the network can achieve a more stable and reliable consensus mechanism, reducing the disparity in reputation scores among nodes.

- Use Case: Secure Patient Data Sharing in Healthcare

To illustrate the effectiveness of BR-PBFT, consider a secure patient data-sharing scenario involving hospitals, insurance companies, and pharmacies. When a patient visits a hospital, their EHRs need to be shared securely with relevant entities while maintaining privacy and integrity.

- Transaction Initiation: A hospital submits a request to share a patient’s encrypted medical records with an insurance provider for claims processing.
- Node Validation and Pre-Processing: The request is validated by participating blockchain nodes to ensure the data is complete and authorized.
- Reputation-Based Leader Selection: A node with a high Beta Reputation Score is probabilistically selected as the leader using VRF, ensuring trust and fairness.
- Consensus Execution: Nodes verify the authenticity of the transaction through the BR-PBFT process (Pre-Prepare, Prepare, Commit phases).
- Finalization and Blockchain Commitment: Once consensus is reached, the transaction is stored immutably on the blockchain, ensuring tamper-proof patient data sharing.

By leveraging BR-PBFT, healthcare organizations ensure that only trusted entities participate in consensus, reducing fraud risks and enhancing data privacy while maintaining high efficiency, make it a state-of-the-art and light-weight method when compared to the existing methods [39].

### 5.2 Contributions to the field

The performance of BR-PBFT was compared with traditional PBFT and SBFT under varying network sizes and Byzantine node conditions. This study makes the following key contributions:

- We propose a light weight consensus model BR-PBFT, an enhanced PBFT variant integrating a refined Beta-based reputation modeling approach for trust assessment.
- We introduced a VRF-based leader selection mechanism, ensuring fairness and decentralization in healthcare blockchain applications.
- We conduct comparative performance evaluations and demonstrate improved efficiency, achieving a 20% reduction in latency and memory consumption by 10% and 15% improvement in computational efficiency over traditional PBFT.
- We provide the analysis to validate security enhancements, showing resistance against Byzantine failures through the incorporation of a robust reputation mechanism.
- Higher Consensus Success Rate: The integration of reputation scoring helped mitigate Byzantine failures, ensuring a higher success rate of transactions.

## 6 Discussion and future scope

BR-PBFT integrates credibility-weighted Beta reputation into PBFT and adds a lightweight VRF to keep leader selection fair and unpredictable. Our focus is the consensus layer—reputation-driven shortlist, VRF-scored leader, PBFT commit—while the protocol remains content-agnostic: applications submit a small JSON anchor (hash, URI, issuer, timestamp, policy) and the actual records stay off-chain. This design promotes trusted participation, limits malicious behavior, and delivers transparent, final, and auditable transactions for healthcare networks.

The current implementation simplifies Bayesian inference by using the mean of the Beta binomial distribution to calculate reputation scores. While it does not explicitly involve full Bayesian updating, it captures the essence of Bayesian reasoning by dynamically adjusting parameters *α* and *β* based on positive and negative feedback. This approach ensures computational efficiency, making it suitable for real-time applications in healthcare blockchains. The results of the BR-PBFT implementation highlight significant improvements over traditional PBFT and SBFT in terms of trust management, efficiency, and security. The key findings of this study include:

- Improved Trust and Security: The integration of the Beta Reputation Score (BRS) effectively prevents malicious nodes from influencing consensus decisions, reducing the risk of Sybil attacks and Byzantine failures.
- Enhanced Efficiency: The optimized leader selection process using Verifiable Random Functions (VRF) reduces consensus overhead, improving transaction throughput and reducing latency by approximately 20
- Fair Leader Selection: Unlike traditional PBFT, where the leader is fixed or rotates predictably, BR-PBFT ensures fairness through cryptographically verifiable randomness, minimizing centralization risks.
- Scalability Considerations: The ability to dynamically select high-reputation nodes reduces computational burden and communication overhead, making BR-PBFT more scalable for large-scale healthcare applications.

Despite these advantages, some challenges remain. The system relies on periodic reputation updates and per-node state, which adds storage and compute overhead. In highly dynamic settings, newcomers may take time to build sufficient reputation, limiting early participation. Moreover, the current design uses binary feedback only (positive/negative). While simple and auditable, this coarse signal reduces granularity and may require more rounds to separate borderline behavior; extending to multi-level or bounded real-valued ratings is a natural direction for future work.

### 6.1 Future scope

Future work will explore integrating more advanced Bayesian techniques, such as incorporating multinomial distributions and confidence intervals, for a comprehensive probabilistic reputation framework. Furthermore, exploring the applicability of Beta Reputation PBFT with VRF in other domains beyond healthcare, such as finance, supply chain management, and government, presents exciting opportunities for extending the benefits of decentralized trust mechanisms to diverse industries and use cases. Therefore, future research can extend this work by:

- Exploring adaptive Dirichlet reputation models that dynamically adjust to real-time behavioral changes.
- Investigating privacy-enhancing techniques, such as Zero-Knowledge Proofs (ZKP), to enhance transaction confidentiality.
- Deploying BR-PBFT in real-world healthcare systems to validate its efficiency and security in live environments.
- Combining reputation-based PBFT with lightweight Proof-of-Stake (PoS) mechanisms to further optimize performance.

Overall, BR-PBFT offers a robust framework for trust-driven consensus in healthcare, paving the way for more secure and efficient blockchain applications in the industry.

## 7 Conclusions

The successful integration of blockchain technology in healthcare depends on robust consensus mechanisms to ensure data integrity, reliable transaction processing, and stakeholder trust. Among existing algorithms, SPBFT has proven effective in handling Byzantine faults, especially in networks with a limited number of nodes. To enhance security and efficiency, we introduce BR-PBFT, which integrates a beta reputation scoring system and VRF. This innovation improves latency, increases throughput, and reduces computational complexity. Our preliminary evaluations show that BR-PBFT significantly outperforms SPBFT in resource efficiency, maintaining lower CPU and memory usage as node numbers grow, making it a more scalable solution for resource-constrained environments. As the healthcare industry evolves, adopting advanced consensus mechanisms like BR-PBFT will be essential to address the complexities of sensitive healthcare data and transactions. By strengthening trust and reliability in decentralized healthcare systems, BR-PBFT with VRF has the potential to drive transformative change and accelerate blockchain adoption in healthcare and beyond.

Our study presents BR-PBFT, a reputation-integrated variant of PBFT, which significantly enhances the efficiency and trustworthiness of blockchain-based healthcare applications. Experimental results demonstrate a 20% reduction in latency and memory consumption along with 15% improvement in computational efficiency compared to traditional PBFT. Future work will focus on deploying BR-PBFT in a real-world healthcare data-sharing platform, optimizing reputation calculation methods, and exploring hybrid models integrating machine learning-based trust management.
